# The digital revolution in phenotyping

**DOI:** 10.1093/bib/bbv083

**Published:** 2015-09-29

**Authors:** Anika Oellrich, Nigel Collier, Tudor Groza, Dietrich Rebholz-Schuhmann, Nigam Shah, Olivier Bodenreider, Mary Regina Boland, Ivo Georgiev, Hongfang Liu, Kevin Livingston, Augustin Luna, Ann-Marie Mallon, Prashanti Manda, Peter N. Robinson, Gabriella Rustici, Michelle Simon, Liqin Wang, Rainer Winnenburg, Michel Dumontier

**Keywords:** phenomics, phenotypes, acquisition, interoperability, semantic representation, knowledge discovery

## Abstract

Phenotypes have gained increased notoriety in the clinical and biological domain owing to their application in numerous areas such as the discovery of disease genes and drug targets, phylogenetics and pharmacogenomics. Phenotypes, defined as observable characteristics of organisms, can be seen as one of the bridges that lead to a translation of experimental findings into clinical applications and thereby support ‘bench to bedside’ efforts. However, to build this translational bridge, a common and universal understanding of phenotypes is required that goes beyond domain-specific definitions. To achieve this ambitious goal, a digital revolution is ongoing that enables the encoding of data in computer-readable formats and the data storage in specialized repositories, ready for integration, enabling translational research. While phenome research is an ongoing endeavor, the true potential hidden in the currently available data still needs to be unlocked, offering exciting opportunities for the forthcoming years. Here, we provide insights into the state-of-the-art in digital phenotyping, by means of representing, acquiring and analyzing phenotype data. In addition, we provide visions of this field for future research work that could enable better applications of phenotype data.

## Introduction

Phenotypes are broadly defined as observable characteristics of organisms and have gained great importance since the discovery of the causative relationship between a given underlying genetic mechanism (e.g. gene expression levels, mutations) and its phenotypic manifestation. Subsequently, diverse initiatives have focused on developing and curating resources that capture this causal relationship at multiple levels and in the context of multiple organisms. Examples include, but are not limited to the Online Mendelian Inheritance in Man (OMIM) database [1], the Mouse Genome Informatics database (MGD) [2], FlyBase [3] and the Zebrafish Model Organism database (ZFIN) [4].

The increasing development and exploitation of phenotypes has led to a varied range of applications: identifying disease genes [5–9] and characterizing functionally yet unclassified genes [10–12], repurposing drugs [13, 14], pharmacogenomics [15–17] and pharmacovigilance [18], as well as solving evolutionary questions [19, 20].

The goal of this review is to synthesize the state-of-the-art in the past 10 years of Phenome Research, and hence provide a unique and broad access point for those interested in studying topics in specific areas of phenomics. The areas covered include both phenotype data evolving from biological experiments and data needed in a clinical environment. This work also presents visions for the coming years in phenomics research, as it derives a series of open challenges, based on input collected from the community. We note here that the work addressed in this article focuses on computational phenotyping, i.e. the collection, representation and processing of phenotypes in a computer-interpretable format.

**Figure 1.**
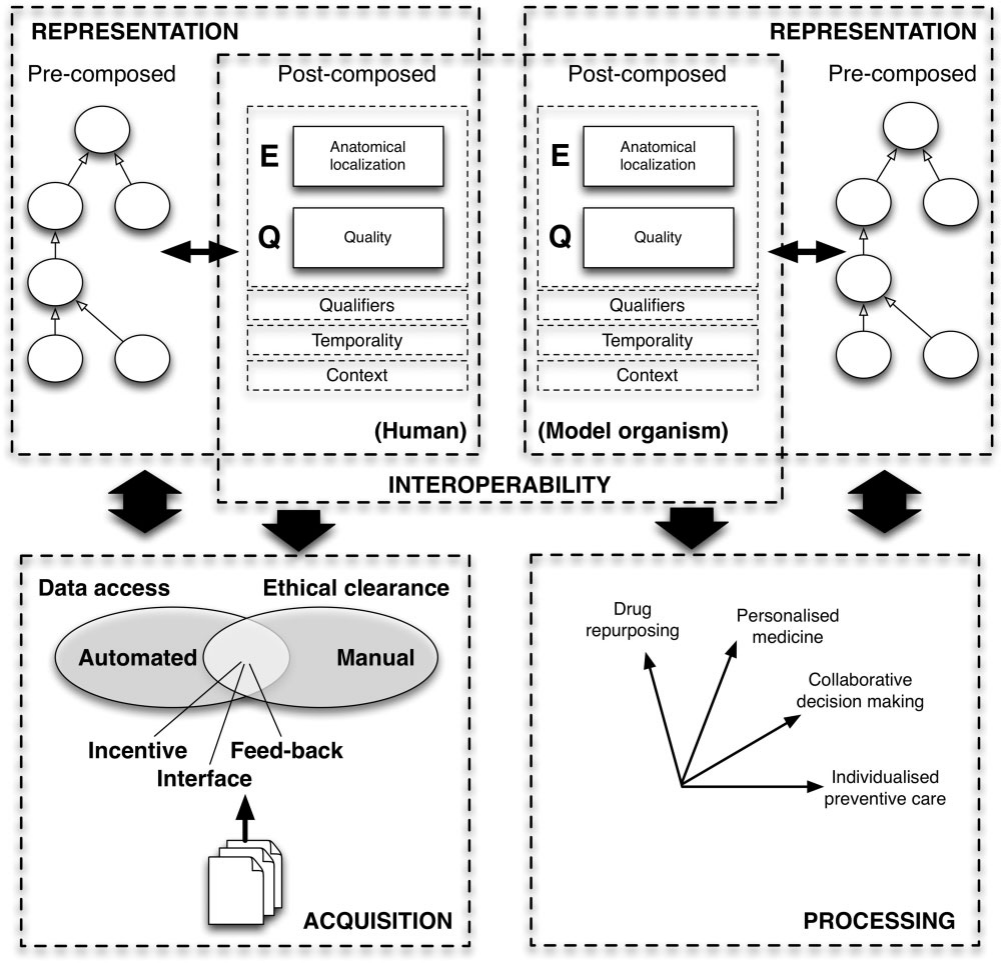
The four dimensions of the phenotype development phases. Representation: Subject to the underlying domain and goal, phenotypes may be represented at different granularity levels. Interoperability: Existing ontologies and vocabularies externalize domain-specific phenotype knowledge at different levels of granularity. Acquisition: Capturing and documenting phenotypes in any representational format can be achieved manually (via curation) or automatically (via text mining). Processing: Representing and capturing phenotypes in a structured manner (a form that also enables interoperability) has led to their application in a large variety of domains. The arrows denote direct points of connection between the several phenotyping dimensions. Note that this figure only serves as illustration of the interplay of the four dimension and, thus, is not aimed at comprehensiveness (e.g. interoperability could also be achieved with a mapping instead of EQ statements).

To enable a structured navigation of the field, we map the content of the review onto the four conceptual dimensions of phenomics, considered from a computational perspective (depicted in Figure 1 ): representation, interoperability, acquisition and processing. These four dimensions have been identified and described in an earlier review [21] and are used for simplicity here. Representation focuses on semantic modeling and aspects of knowledge capturing. Interoperability, an orthogonal dimension to representation, aims to facilitate intra- and interspecies phenotype mappings. Acquisition refers to the transformation of the raw data into semi-structured or structured phenotype representations. Processing (or application) uses externalized phenotypes to address fundamental or specific challenges, from variant prioritization and diagnosis to individualized preventive care or drug repurposing.

Each of these dimensions encapsulates a multitude of aspects that reflect in their aggregated form the intrinsic complexity of phenotypes. For example, subject to the underlying domain, the granularity of the representation of phenotypes may differ depending on their application. While biologists capture data that may be too detailed for clinical applications, clinicians need solid and thoroughly supported evidence to be able to test hypotheses derived from biological experiments. Furthermore, phenotypes may be defined and represented in the context of different organisms, e.g. decreased bone mineral density (MP:0000063) in the Mammalian Phenotype Ontology (MP) [22] and Osteopenia (HP:0000938) in the Human Phenotype Ontology (HPO) [23], even though they may share an underlying goal from a translational perspective, such as the documentation of a certain gene function. This leads to the need for achieving cross-species interoperability to enable integrated processing. Finally, the phenotype acquisition process poses its own challenges, i.e. technical (process automation or interface usability), social (incentive) and ethical (privacy). In the following we discuss in depth each of the dimensions introduced above. A summary of all the resources provided in this manuscript is provided in Table 1.

**Table 1.** Summarizes all resources mentioned throughout the manuscript, together with their URL and reference (where applicable)

Resource	Link	Reference
Online Mendelian Inheritance in Man database	http://omim.org/	[1]
Mouse Genome Database	http://informatics.jax.org/	[2]
FlyBase	http://flybase.org/	[3]
Zebrafish Model Organism Database	http://zfin.org/	[4]
Mammalian Phenotype Ontology	http://www.berkeleybop.org/ontologies/mp/	[20]
Human Phenotype Ontology	http://purl.obolibrary.org/obo/hp.obo	[21]
London Dysmorphology Database	http://www.lmdatabases.com/	[23]
Gene Ontology	http://geneontology.org/	[24]
Phenotypic quality and Trait Ontology	http://purl.obolibrary.org/obo/pato	[25]
OrphaNet	http://www.orpha.net/consor/cgibin/index.php	[26]
PharmGKB https://www.pharmgkb.org/ [27]		
Zebrafish Anatomical Ontology	http://purl.obolibrary.org/obo/zfa	[28]
International Mouse Phenotyping Consortium	http://www.mousephenotype.org/	[29, 30]
IMPReSS	https://www.mousephenotype.org/impress	
Phenote	http://www.phenote.org/	
PhenoTips	https://phenotips.org/	[31]
MetaMap	http://www.nlm.nih.gov/research/umls/implementation_resources/metamap.html	[32]
NCBO Annotator	https://bioportal.bioontology.org/annotator	
cTakes	http://ctakes.apache.org/	[33]
ShARE/CLEF 2013	https://sites.google.com/site/shareclefehealth/data	[34]
DeepPhe	http://cancer.healthnlp.org/	
Bio-LarK	http://bio-lark.org/	[35]
PhenoMiner	https://sites.google.com/site/nhcollier/projects/PhenoMiner	[36]
Unified Medical Language System	http://www.nlm.nih.gov/research/umls/	[37]
Unified Medical Language System Metathesaurus tool	http://www.nlm.nih.gov/pubs/factsheets/umlsmeta.html	[38]
UberPheno	http://purl.obolibrary.org/obo/hp/uberpheno/	[39]
SNOMED CT	http://www.nlm.nih.gov/research/umls/Snomed/snomed_main.html	[40]
AgreementMaker	http://agreementmaker.org/	[41]
Zooma	http://www.ebi.ac.uk/fgpt/zooma/	
SIDER	http://sideeffects.embl.de/	[14]
AVAToL	http://avatol.org/	[17]
PhenoScape	http://phenoscape.org/	[18]
ORCID	http://orcid.org/	
ResearcherID	http://www.researcherid.com	

## State-of-the-art phenome research

### Representation

A phenotype can be any observation of a normal or abnormal state of an anatomical, physiological or biochemical property of an organism. While phenotypes in a biological domain are recorded as results from biological experiments, phenotypes in a clinical domain are used to report the assessment of patients. Phenotypes span from the molecular level to the organism level [42]. To enable the other three dimensions to reach their full potential, a representation is needed that is well understood by humans, and at the same time, computer-readable and hence amenable to computational analyses. Such a representation does not only have to cover normal and abnormal phenotypes within a species, but also has to facilitate the bridging across species and enable integration of heterogeneous data at different levels of granularity.

From a computational perspective, phenotypes take diverse representations: (i) free-text descriptions—e.g. as part of the OMIM disease presentations, (ii) vocabularies—e.g. the clinical synopsis in OMIM, or the London Dysmorphology Database terminology [25], and (iii) ontologies—i.e. vocabularies augmented with domain-specific relationships—e.g. HPO or MP. While free text descriptions support a better human understanding, they limit the possibilities for automated data analysis [43]. With the abundance and ever-increasing amount of data, the ultimate goal is to build a uniform and consistent computer-readable representation, one that enables a seamless collection and integration of phenotypes recorded in biological studies, as well as in a clinical environment. Ideally, this uniform, global representation would also account for both qualified and quantified data and enable flexible conversion where possible. In cases where conversion is not possible, this universal representation would have to be extended with mappings.

#### Ontologies to represent phenotypes

**Figure 2.**
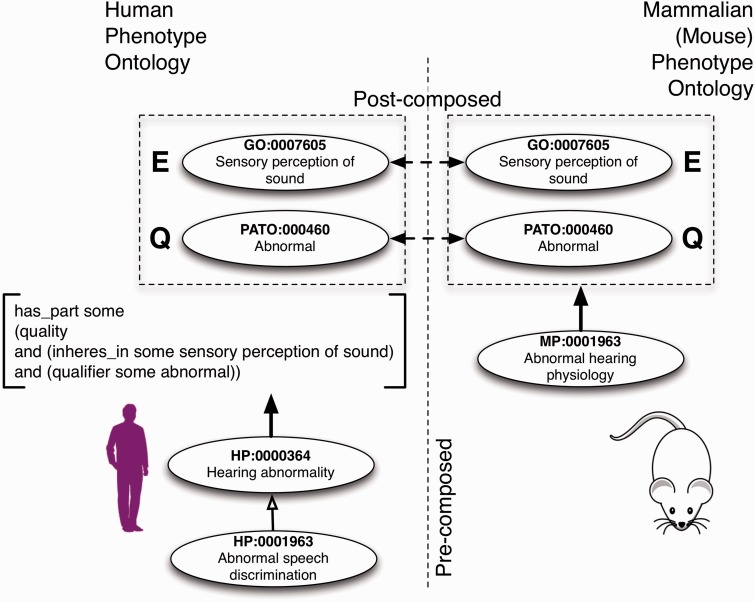
To date, phenotypes have mostly been captured and defined using a pre-composed and/or a post-composed representation. A pre-composed representation assumes the definition of a phenotype as a monolithic concept—a concept that captures the essence of the phenotype semantics. The post-composed representation decomposes the phenotype into an Entity–Quality pair, with its individual components being mapped to appropriate ontological concepts. In this case, the phenotype semantics is denoted by the compositional property of the pair. The transition between pre-composed and post-composed is realized via logical axioms. Both forms of representation have been successfully applied across different species.

Currently, the field consists of a varied set of vocabularies and ontologies that support, in various forms, the abovementioned goal. In particular, driven by the wide adoption from the biomedical community, ontologies have become the de facto standard for representing phenotypes. To achieve the goal to its full extent, the community has followed two complementary approaches for modeling and integrating phenotype data: a pre-composed and a post-composed representation (see Figure 2). The pre-composed approach treats each phenotype as an atomic entity, using individual expressions most suitable to general human understanding. For example, an ontology adopting this representation consists of concept definitions like ‘erythrocytopenia’ or ‘deficiency of red blood cells’ / ‘deficiency of erythrocytes’. These concepts are easily understood by humans and also facilitate computational analysis.

The post-composed representation uses elementary phenotypic units from existing ontologies to compose specific complex phenotypes. One mechanism to postcompose phenotypes is the entity-quality (EQ) statement approach [24, 44]. For example, instead of defining ‘erythrocytopenia’ as an atomic concept, this approach represents the meaning of the phenotype by linking the quality ‘deficiency’ with the anatomic entity ‘red blood cells’. This link is then captured via a logical axiom using concepts introduced by existing ontologies, such as the Gene Ontology (GO) [26] and the Phenotypic quality and Trait Ontology (PATO) [24]. The caveats of the postcomposition result from the development overheads in building post-composed statements. Additionally, a number of pre-composed phenotype ontologies still need to be transformed into a post-composed representation.

#### ‘Normal’ and ‘abnormal’ phenotypes

Some of the existing phenotype representations focus on deviations of phenotypes (i.e. their status or quality, from a reference phenotype) [27]. The reference phenotype in the case of model organisms could be either the wild type of the organism or a specific strain from which the mutation has been generated. In the best case, the phenotype representations form the core that enables interoperability of different data repositories, possibly covering different organisms and being collected with different aims in mind. The quality of the phenotypic resource, i.e. the consistency of the phenotypic definitions, the overall structure of the phenotype semantic resource and in particular the completeness of the electronic resource, holds the key to enabling efficient data analysis, interpretation and decision support.

To extend beyond representing ‘abnormal’, Shimoyama *et al.* have extended the representation of phenotypes to also incorporate environmental factors as well as methods used to measure the phenotype [45]. The developed ontologies have been used to annotate both rat (http://rgd.mcw.edu/wg/physiology) and human data (http://cover.wustl.edu/Cover/). Furthermore, the suggested framework allows for the annotation of quantified phenotypes (e.g. ‘blood sugar levels > 8.5 mmol/L’) instead of ‘abnormal / increased blood sugar levels’. While this way of representation could be used to represent the reference phenotype, a data curator is needed to provide this information. Conversions from the different states of ‘normal’, e.g. similar tests in different species, are not automatically available.

#### Representing qualitative and quantitative phenotypes

Other desiderata for phenotype representations are focused on the exploitation of ontologies for efficient propagation of experimental findings in basic phenomics research into the clinical domain and improved research efficiency in both domains (translational medicine). Consequently, phenotype descriptions have to meet clinical needs and cover those diseases that are most relevant to the clinical context. Experimental phenotypic descriptions are detailed and reflect the experimental setup, whereas clinical descriptions suffer from time constraints and thus tend to lack observational detail. Furthermore, experimental and clinical phenotypic descriptions may be organized at diverse levels of granularity and may be biased toward a specific perspective. For example, experimental findings provide the opportunity to capture and represent quantitative traits (e.g. ‘blood sugar > 8.5 mmol/L’), which may require adaptation into qualitative terms (‘high blood sugar’) for clinical purposes. Similarly, from a diagnosis perspective, one may require a complete and individualized view over the phenotypic profile, which may include degrees of severity [28] and longitudinal phenotypes [29], hence adding to the overall complexity of the representation.

#### Representation of phenotypes: summary

The resources for representing phenotypes have reached a point where they are able to provide a solid and rich foundation for building advanced acquisition and processing mechanisms. Open challenges still exist, e.g. modeling degrees of severity, normal states or negation (i.e. explicitly mentioning the absence of an abnormality) or mapping quantitative traits to qualitative concepts to provide deep knowledge capturing methodologies.

### Acquisition

**Figure 3.**
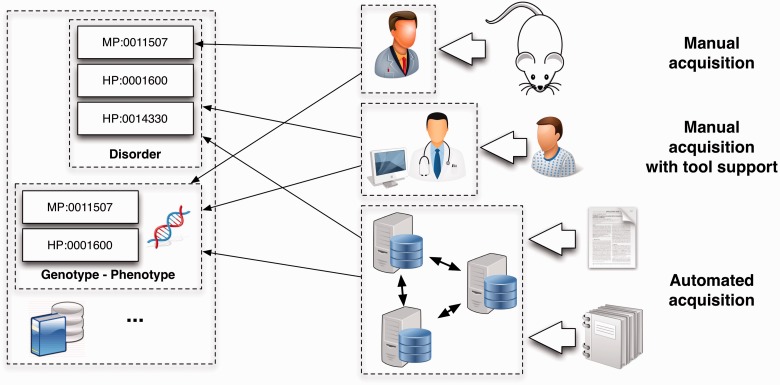
The increasing amount of data made available over the course of the past years have rendered manual phenotype curation impractical. While automating the process is in principle the only viable solution, it possesses its own plethora of technical challenges. These include, among others: (i) boundary detection, i.e. identifying the exact span of text that represents a phenotype candidate; (ii) disambiguation and alignment, subject to the desired level of granularity and the underlying knowledge source; and (iii) interpretation, which covers lack of context, hedging or negation.

Acquisition involves the collection and storage of phenotype information from various resources (see Figure 3), such as OMIM or OrphaNet, a rare disease database [30]. While some of these resources are mainly built through manual curation, e.g. MGD, others rely already on (semi-)automated preprocessing to enhance curator throughput. For example, PharmGKB [46] uses an automated classification system to determine relevant publications and extract gene–drug relationships that are then provided to curators for verification [31, 47].

#### Manual acquisition of phenotypes

Manual acquisition of phenotypes can be done either by curation of the literature or by direct submission from investigators. These two main modes are used to annotate model organism data with phenotype observations and their conceptual descriptions. In the case of MGD, curators provide standard phenotype descriptions from MP along with supporting evidence. ZFIN similarly provides phenotypic data with evidence coming from manual curation and individual investigator contributions using the Phenote software (http://www.phenote.org/). Phenote allows description of phenotypes in an EQ format, which makes use of any ontology in the Open Biological and Biomedical Ontologies [48] format, including PATO, the Zebrafish Anatomical Ontology [32] and GO.

The International Mouse Phenotyping Consortium (IMPC) [33, 49] has applied phenotype encoding standards [50] through a set of standard operating procedures (SOPs; as defined in IMPReSS: https://www.mousephenotype.org/impress) for recording high-throughput phenotype measurements in the lab. Each of the SOPs describes not only the experimental setup for the measurement of the required parameters, but also the ontology annotation this test may induce. For example, the SOP designed to assess the grip strength of a mouse includes the suggestion of the MP term ‘abnormal grip strength’ (MP:0001515).

In addition to Phenote mentioned above, one example of a system that was designed specifically for phenotype capture in a manual mode is PhenoTips [51]. This open-source system assists clinicians to record phenotypic profiles for patients with rare genetic disorders using HP and OMIM, potentially allowing for diagnosis and comparative phenotype analysis.

Discovering evidence for the causes of human disorders and providing treatment are common goals across the clinical and scientific communities. However, the understanding of phenotypes has traditionally been different between the two communities. Clinicians generally consider phenotypes to be aberrations, i.e. deviations from normal morphology, physiology or behavior [52], while scientists working on biological experiments, such as mutation experiments, have adopted a more pragmatic definition of a selective profile of all the observable characteristics of an organism. This division stems in part from a focus on the overt expression of the syndromes themselves [53] on the one hand and on the pathway from syndrome to gene expression on the other. Both are crucial to understanding the complex nature of disorders, as Sabb *et al.* point out [53]. This difference is reflected in the type of data that each community creates and the systems that have been built to support data capture by each.

#### Semi-automated and automated phenotype acquisition

With the increasing amount of data that is published on a day-to-day basis, manual approaches for data curation become more and more time-demanding and costly, so that computer assistance in screening (document retrieval) and preparing data (information extraction) is unavoidable. The degree to which computer assistance is enabled determines whether the method is semi-automated or automated. While in a semi-automated setting a curator manually verifies the extracted data, in an automated setting no manual input is required. However, given the absence of manual verification and the current state-of-the-art in text processing, the data generated with an automated method may contain some incorrect data.

The structural and semantic complexity of phenotype terms, coupled with the scale and changing nature of literature-based phenotype descriptions, makes a traditional fully manual acquisition approach difficult to sustain, leading to potential duplication, inconsistency and sub-optimal coverage. This has created a growing interest in text/data mining techniques. A diverse and growing research community is evolving that aims to exploit biomedical natural language processing for the extraction of structured data from free-text and its annotation with the semantic resources that already exist. Although not specifically aimed at phenotypes, knowledge brokering tools such as MetaMap [34], the NCBO Annotator and the Apache cTAKES [54] have all been widely used for concept annotation of text to biomedical ontologies and could be used to yield these building blocks. The issue of customizing these generic tools to the extraction of phenotypes in specific disease domains is a key challenge.

Extraction of structured information from electronic health records (EHRs) has a long history of research, e.g. [35, 36, 38, 55]. Progress has been hampered by the balance that needs to be drawn between respecting patient privacy and the need for data to develop comparable gold standards. In the past few years, several initiatives have led the way in making available anonymized collections of EHRs, e.g. Informatics for Integrating Biology and the Bedside (i2b2) [56] and recently ShARE/CLEF 2013 [57]. These tasks aim to identify entities of clinical interest including medical problems, tests and treatments. While neither of these data sets explicitly annotates phenotypes, these entities are highly relevant to phenotype acquisition. Fu *et al.* suggested an annotation scheme to capture phenotypes for chronic obstructive pulmonary disease in EHRs, which has been implemented in Argo [58] to annotate a corpus of 1000 clinical records [59]. Furthermore, the newly launched DeepPhe project (http://cancer.healthnlp.org/) focusses on phenotypes relevant in the cancer genomics domain.

Using the scientific literature as a source, several groups have been active in developing approaches explicitly for phenotypes. These include the Bio-LarK system, which has been applied to skeletal dysplasia [39] and the PhenoMiner system, which has been applied to the cardiovascular and autoimmune systems [60]. Work by Khordad *et al.* [37] has looked at a more mixed domain using the Unified Medical Language System (UMLS) Metathesaurus tool [40]. Ongoing challenges in processing EHRs are: descriptive naming (e.g. typical course face, heterogeneous ECG abnormalities), disjoint phenotype mentions (e.g. blood pressure was observed to be elevated) and coordinated terms (e.g. slow healing and excessive scarring). Harmonization to existing ontologies presents an additional layer of challenge in deciding how to align phenotype mentions that are more or less specific than extant concepts and how to provide sufficient evidence for human curators.

While automated methods are not as thorough as curators, they overcome some of the bottlenecks experienced with manual curation, e.g. high time consumption and low throughput. In general, there is a trade-off between thoroughness (precision) and the amount of acquired data (recall) returned as results from these methods, i.e. automated methods may not return all relevant results and may return some incorrect results.

#### Acquisition of phenotypes: summary

The acquisition and harmonization of phenotypes is an ongoing challenge to be met using evidence from a variety of sources (e.g. EHRs, scientific literature, clinical reports). The key issue for automated approaches involving natural language processing support is to identify and resolve lexical, syntactic and semantic heterogeneity.

### Interoperability

The interoperability dimension of phenomics research focuses on making all the available phenotype data integrable with other data sources, e.g. diseases or results from genome analyses. The overarching goal of interoperability is to facilitate translational research and biological discoveries [21]. Current and past work falling into this dimension can be summarized as standardization efforts, alignment of phenotypes within and across species and mapping to other resources. Challenges arise from the many levels of complexity phenotypes can span [42] as well as the development of multiple, and mostly disparate, reporting schemes [22, 23, 41].

#### Interoperability through semantic layers

A prerequisite for interoperable phenotype resources is a semantic layer that spans across the resources applied and allows to keep the consistency and specificity contained in each of the resources. For example, despite standardization efforts such as the Minimal Information for Mouse Phenotyping Procedures [61], the existing landscape of mouse phenotype resources is not fully interoperable and hard to manage [62]. A similar scenario is seen in hospitals where different wards use disparate ways of describing a patient. As a consequence, the data for a patient cannot readily be used for further analysis, preventing potential holistic treatment opportunities. However, the need for standardized reporting has been recognized and is implemented through SOPs in the IMPC [33, 49, 50].

While historically there have been different phenotype representations for different species, such as the human, mammalian, fly and worm phenotype ontology (see 2.1 representation), EQ statements (see Figure 2) have been suggested to integrate phenotypes across different species [24, 44]. In addition, an amendment to the existing EQ statements was suggested to make them interoperable with anatomy and physiology ontologies [63] to extend the links across the different layers of complexity. To make the annotation for three different species (human, mouse and zebrafish) more accessible, Köhler and authors made the UberPheno ontology publicly available [64].

#### Interoperability achieved through mappings (alignment)

Further to the representation of phenotypes with EQ statements, manual [65] and automated methods are in progress to align different pre-composed semantic representations. One example is UMLS [66] that combines over 180 vocabularies, terminologies and ontologies, such as SNOMED CT [67]. The integration of new resources into UMLS is semi-automated. Conflicts between concepts from newly added resources and concepts already in UMLS are manually resolved to ensure a high-quality alignment of all the incorporated terminologies, vocabularies and ontologies.

As an alternative to manual and semi-automated solutions, tools exist that provide fully automated alignments between ontologies, such as AgreementMaker [68] and Zooma (http://www.ebi.ac.uk/fgpt/zooma/). While AgreementMaker takes lexical matching and ontological features into account, Zooma uses phonetic matching algorithms for the alignment. In many cases, the resulting alignments often associate one term with multiple concepts (1:n mapping). Bottlenecks in the automated alignment are caused by species-specific jargon [69, 70] and by phenotypes that only exist in one of the species and not the other.

In addition to the alignment of multiple resources, mappings are required that would facilitate the integration of diverse resources spanning across the different layers of complexity of an organism. While phenotypes in model organisms have been assigned to genes as well as to specific models (determined by allele and background in addition to gene, e.g. MGD), in human, mostly inheritable diseases have been annotated. However, if we were to follow the pathway from the modified gene to the observed phenotypes, a mapping between pathways and phenotypes would also be required [71]. As mentioned above, some integration with other resources has been achieved, e.g. with anatomy and physiology ontologies, but a much larger coverage is required to unlock the full potential of knowledge discovery using phenotype data.

#### Interoperability of phenotypes: summary

The need for unified and integrable representation of phenotypes has been recognized, and projects are underway to improve the current situation. However, there is still a huge amount of legacy data that need to be dealt with.

### Processing

Processing (or application) is concerned with the use of phenotype data that have either been reported in structured form or extracted with, for example, text mining methods (see acquisition methods of phenotypes). Subject to the target domain, the usage of phenotype data can be classified into four broad categories: (i) clinical research (diagnosis, prognosis, patient match-making, variant prioritization, personalized medicine, drug side effects); (ii) study of genome–phenome interactions to advance the understanding of disease causation or achieve personalized therapies; (iii) cross-resource consistency analysis; and (iv) evolutionary research. It is worth noting that in most cases phenotype data have been used in a cross-species context, hence transforming the interoperability dimension into a first-class citizen, rather than an application scenario.

#### Application of phenotypes to study the origins and pathology of diseases

The application area of clinical research consists of a varied set of specific goals, which represent in practice coherent research streams on their own. Phenotypes have been used as unique source of data, for example, for disease prediction [72, 73], mining key disease characteristics or characteristic phenotypes [16, 17, 74] or patient match-making [51]. Furthermore, phenotypes have been used to support the understanding of the genetic mechanism of diseases via direct association with genotype data [5, 9, 12], as a mapping bridge across species data [7, 8] and in conjunction with the entire set of OMICS data [75] or to improve variant prioritization for accurate diagnosis [76, 77].

More recently, phenotypes have played a major role as a discovery agent in large-scale genome-wide association studies [78–80]. In particular, projects such as the 100 000 Genomes Project (http://www.genomicsengland.co.uk/the-100000-genomes-project/) as well as the eMerge Network (https://emerge.mc.vanderbilt.edu/?page id=58) aim to support the area of Pharmacogenetics by linking data from EHRs to sequence information from patients to improve diagnosis and treatment. Similarly, Phenome-wide Association Studies (PheWAS) [81–83] allow for the identification of genes that possibly implicate a disease and can provide provenance for results determined through Genome-wide association studies.

#### Phenotypes and ethnicity

Generic phenotypes have an immense potential, which has been only recently discovered and exploited. For example, ethnic differences have been shown to explain the optimal states of the human blood glucose levels—expressed via the relationship between insulin sensitivity and insulin response [84]. Similarly, when used in the context of a shared genetic architecture, such data validated the existence of statistically significant relationships between the platelet count and alcohol dependence, or between the alkaline phosphatase level and venous thromboembolism [85]. This application area is characterized by a specific set of challenges, emerging from the novel combination of numeric data (from tests and measurement), abnormalities and standard traits. One important and still unsolved problem in the processing of phenotypes is the lack of alignment between measurement representations and phenotypic abnormalities, as well as the ability of representing states of normality and longitudinal phenotypic data resulting from tests and measurements.

#### Phenotypes in drug repurposing

Phenotypes expose the effects of drug treatments, and hence, enable the study of their general effects, the relation between dosage and effects, as well as the interaction between drugs. Existing literature on this topic maps perfectly onto these three aspects. Phenotypes, as adverse drug reactions, have been modeled and captured, as early as 2010, by the SIDER initiative [14] and have been used to investigate the causal relationship between dosage and effect by [15]. In an exercise that combines large-scale acquisition and processing, LePendu *et al.* have used phenotypes as indicators of adverse drug reactions, as well as signals of adverse events associated with drug–drug interactions [18]. Finally, with the increasing curation, adoption and use of cross-species phenotype data, it has been shown that an effective mapping between model organisms and drug effect profiles based on similarity can be applied to successfully suggest candidate drug targets [13].

#### Phenotypes in evolutionary studies

In this context, phenotypes have been used to understand patterns of diversification and to gain additional knowledge on trait evolution. Two particular initiatives have focused on this aspect. The AVAToL project [19] represents a collaborative and multidisciplinary effort that combines text mining, image analysis and the wisdom of the crowds to discover and document species phenotypes. Their ultimate goal is to advance phylogenetics research and to enable a faster and more accurate construction of the Tree of Life. The Phenoscape knowledge base [20], on the other hand, integrates phenotype data acquired on over 2500 teleost fishes with structured phenotype data from zebrafish genes to infer candidate genes that explain phenotypic variety, and hence, enable the formulation of evolutionary/devolutionary hypotheses.

#### Processing of phenotypes: summary

The quality and range of phenotype applications are curbed only by the quality and availability of the underlying data. Limitations arise, for example, from the missing or incorrect cross-species phenotypes alignment, either owing to their foundational representation or owing to inconsistencies in the level of granularity. Similarly, challenges are encountered when representing and acquiring the more profound dimensions of phenotypes, including degrees of severity, use of ambiguous expressions or temporality (in a longitudinal data sense), which then hamper the development of complex solutions.

## A future perspective of phenome research

For the entire field of Phenomics to advance, challenges have to be overcome at the universal level, as well as at the level of the individual dimensions of phenome research (representation, acquisition, interoperability and processing). The most important universal challenge is the lack of a shared understanding of what a phenotype is among all scientists working with phenotype data. This includes (computational) biologists, regardless of the research questions and/or animal model they are working on, as well as clinicians working in all different areas of human medicine. To reach a common understanding, reporting standards and guidelines need to be derived that facilitate communication across domains as well as large-scale computational analysis.

Furthermore, universal agreement has to be found as to what additional aspects of phenotypes are relevant and have not or only sparsely been accounted for in any of the four domains. Such aspects include types of measurements, provenance, evidence and time. To overcome the social barriers to adoption, some sort of credit assignment mechanism, akin to citation, will be necessary to track the usage of ‘good’ and reusable phenotypes. Existing author tracking systems such as ORCID (http://orcid.org/) and ResearcherID (http://www.researcherid.com/) can probably be reused to document authorship of phenotype models, and track usage as well as provenance.

Another important universal issue to overcome is the recording of ‘normal’ phenotypes. Currently, phenomics in the area of disease gene discovery is geared toward the collection and analyses of ‘abnormal’ phenotypes, e.g. those resulting from diseases or gene modifications. However, the derivation of an ‘abnormal’ phenotype is always the result of a comparison with what is considered to be ‘normal’, which is collected only in some cases [45]. The record of more ‘normal’ phenotypes would allow for more fine-grained analyses and the investigation of causal relationships between different conditions, e.g. in cases of comorbidity.

From a representational point of view, the ultimate future goal is to overcome the limitations of species- and domain-specific representations and find a universal way of encoding phenotype data, independent from the granularity of the data. In addition, resources need to be built that address so far missing aspects such as evidence or time aspects. For example, a small set of evidence codes have been established as part of GO to provide provenance in gene annotations, but also to provide means for computational analysis to avoid data circularity. Another aspect of phenotypes that has not been integrated yet into the representation of phenotypes are causality and temporality, for example how the phenotypes change over time owing to stimuli in the environment or medication. While at the moment the representations focus on reporting either a temporary snapshot of an individual examined in an experiment or as part of a medical investigation, the current representation models do not allow for the encoding of phenotype changes over time as a result to surrounding stimuli.

As we extend the scope of our joint understanding of phenotypes and adopt ways to represent this understanding, the acquisition of phenotypes has to change, too. Methods have to be developed that can accommodate the recording of additional aspects, such as evidence and time, e.g. when extracting information from the scientific literature. Furthermore, more reliable automated methods are needed that can cope with the complexity of free text in clinical settings as well as reporting mechanisms in wet lab environments, to facilitate high-throughput and overcome the need for time- and cost-intensive manual labor. In addition and as mentioned earlier, the acquisition dimension should also address the collection of ‘normal’ phenotypes in the future to improve the results obtained by processing the phenotype data.

Despite the widespread aims to achieve interoperability and make best use of the integrated resources, there are still challenges that need to be addressed to achieve true interoperability of the existing and newly emerging resources, both phenotype-specific and not. A long-term goal of the dimension of interoperability is direct propagation of experimental findings into prevention and treatment options for patients in a hospital (‘from bench to bedside’). Related to this goal is the aim of personalized treatments, by means of building patient-specific models, integrating phenotype data, that can then be used for simulations of possible treatment outcomes.

In the future, we anticipate a migration toward describing phenotypes as ‘models’ or classifiers that answer a particular question. For example ‘does the patient have pneumonia?’ or ‘does the patient have sepsis?’. The use of phenotyping will then be analogous to the use of classifiers in spam filters; always running in the background and when an incoming sample (a patient record for example) results in a high confidence match, we would automatically receive an alert that the patient is potentially eligible for a clinical trial, is likely to benefit from a certain therapy or is at increased risk for certain complications.

With the improvement in any of the other dimensions, the processing of phenotypes will improve owing to an increase in the quality of data, but at the same time, will require extensions that can cope with additional data available in the future, such as ‘normal’ phenotypes, evidence for phenotype data and causal relationships encoded with time-dependencies. In general, a wide range of support and analysis tools are required to unlock the full potential of phenotype data.

Given the achievements in the past years, we look forward toward an exciting and promising decade of phenomics, with ample opportunities for researchers to get involved and contribute to evolve and shape the emerging landscape.

Key Points

- Over the course of the past decade, phenotype data has become a key factor in analyzing diseases and reporting experimental outcomes.
- Successful applications of phenotypes include the description of experimental outcomes (e.g. the changes in phenotypes owing to gene modifications), computational knowledge discovery (e.g. in determining disease gene candidates) and reporting in clinical environments (e.g. patient monitoring).
- The research field of phenomics can be divided into four main dimensions (presentation, acquisition, interoperability and processing), each of which is dependent, to some extent, on the others.
- While the development of each of the four dimensions is individual, a common understanding and universal guidelines need to be established on how phenotypes are perceived and how they are used; a synchronization of efforts is needed.
- The future of phenomics research holds exciting challenges and has the potential to create a significant impact on the entire biomedical domain.

## Funding

This work was supported by the National Institutes of Health [1 U54 HG006370-01 to A.O., R01 LM011369 and R01 GM101430 and U54 HG004028 to N.H.S., T15 LM00707 to M.R.B., R01-LM008111 to K.L., R01 GM102282 and R01 LM011369 to H.L., U24 CA143840 to A.L., U54HG006370 to A.M., U54 HG008033-01 to M.D.]; the Wellcome Trust [098051 to A.O.]; a Marie Curie experience researcher fellowship [301806 to N.C.]; the National Science Foundation [1207592 to I.G., DBI-1062404 and DBI-1062542 and EF-0905606 to P.M.]; the Bundesministerium für Bildung und Forschung [0313911 to P.N.R.]; the European Community’s Seventh Framework Programme [Grant Agreement 602300; SYBIL to P.N.R]; the Systems Microscopy NoE project [grant agreement 258068 to G.R.]; and the Defense Advanced Research Projects Agency [W911NF-14-C-0109 to K.L.]. T.G. was supported by the Kinghorn Foundation. O.B. was supported by the Intramural Research Program of the NIH, National Library of Medicine. M.S. was supported by the Medical Research Council. L.W. was supported by the Homer Warner Center for Informatics Research of the IHC Health Services. R.W. was supported by an appointment to the NLM Research Participation Program administered by the Oak Ridge Institute for Science and Education through an interagency agreement between the U.S. Department of Energy and the National Library of Medicine.
